# RNA-Seq Atlas of *Glycine max*: A guide to the soybean transcriptome

**DOI:** 10.1186/1471-2229-10-160

**Published:** 2010-08-05

**Authors:** Andrew J Severin, Jenna L Woody, Yung-Tsi Bolon, Bindu Joseph, Brian W Diers, Andrew D Farmer, Gary J Muehlbauer, Rex T Nelson, David Grant, James E Specht, Michelle A Graham, Steven B Cannon, Gregory D May, Carroll P Vance, Randy C Shoemaker

**Affiliations:** 1 Department of Agronomy, Iowa State University, Ames, IA 50011, USA; 2 United States Department of Agriculture-Agricultural Research Service, Plant Research Unit, St. Paul, MN 55108, USA; 3 Department of Crop Sciences, University of Illinois, 1101 West Peabody Dr., Urbana, IL 61801, USA; 4 National Center for Genome Resources, Santa Fe, NM 87505, USA; 5 United States Department of Agriculture-Agricultural Research Service, Corn Insects and Crop Genetics Resources Unit, Ames, IA 50011, USA; 6 Department of Agronomy and Plant Genetics, University of Minnesota, St. Paul, MN 55108, USA; 7 Department of Agronomy, University of Nebraska-Lincoln, Lincoln, NE 68583, USA

## Abstract

**Background:**

Next generation sequencing is transforming our understanding of transcriptomes. It can determine the expression level of transcripts with a dynamic range of over six orders of magnitude from multiple tissues, developmental stages or conditions. Patterns of gene expression provide insight into functions of genes with unknown annotation.

**Results:**

The RNA Seq-Atlas presented here provides a record of high-resolution gene expression in a set of fourteen diverse tissues. Hierarchical clustering of transcriptional profiles for these tissues suggests three clades with similar profiles: aerial, underground and seed tissues. We also investigate the relationship between gene structure and gene expression and find a correlation between gene length and expression. Additionally, we find dramatic tissue-specific gene expression of both the most highly-expressed genes and the genes specific to legumes in seed development and nodule tissues. Analysis of the gene expression profiles of over 2,000 genes with preferential gene expression in seed suggests there are more than 177 genes with functional roles that are involved in the economically important seed filling process. Finally, the Seq-atlas also provides a means of evaluating existing gene model annotations for the *Glycine max *genome.

**Conclusions:**

This RNA-Seq atlas extends the analyses of previous gene expression atlases performed using Affymetrix GeneChip technology and provides an example of new methods to accommodate the increase in transcriptome data obtained from next generation sequencing. Data contained within this RNA-Seq atlas of *Glycine max *can be explored at http://www.soybase.org/soyseq.

## Background

Early hybridization-based studies indicated that the soybean genome has undergone at least one round of large-scale duplication [1]. This finding was supported by analyses of Expressed Sequence Tags (ESTs) [2,3], which suggested an additional duplication event, with estimated times of approximately 14 and 44 mya. The generation of so many duplicated genes likely gave rise to a large number of new, novel and perhaps unique gene functions [4,5]. It is possible to gain insight into their gene function through the exploration of transcriptome data.

With the release of a high-quality draft of the *G. max *genomic sequence [6], we are in a position to significantly improve our understanding of the soybean transcriptome. Previous gene expression studies have been performed using EST sequencing, spotted microarrays and Affymetrix GeneChip technology. These include a study in soybean seed development using laser capture microdissection [7] and studies of the iron stress response in soybean [8]. Other expression atlases have been produced for *Arabidopsis thaliana, Oryza sativa, Lotus japonicus *and *Medicago truncatula *[9-12]. However, array-based methodologies are constrained by prior knowledge of gene sequences. This limits the patterns of gene expression to a subset of the total transcriptional activity in an organism. For instance, the soybean Affymetrix GeneChips used in the Le et al. (2007) study contained sequences that represent 21,790 genes in the Glyma1.01 genome assembly [13,14]. This is less than half of the genes identified as "high confidence" gene models in *G. max *in the Glyma1.01 annotation release, and less than a third of all the predicted genes in *G. max *[6]. As a result, information collected using these GeneChips is incomplete, providing only a fragmented picture of transcript accumulation patterns.

The recent development of next-generation sequencing technology provides information on gene expression independent of genomic sequence knowledge. It also has the advantage of higher sensitivity and greater dynamic range of gene expression than array-based technologies [15-17]. The RNA Sequencing method (RNA-Seq) was originally developed to take advantage of the next-generation Illumina sequencing technology to improve the annotation of the yeast genome and explore its transcriptional expression profile [17]. The RNA-Seq approach was shown to have relatively little variation between technical replicates [16] for identifying differentially expressed genes. This technique has since been applied to several other organisms to answer questions regarding gene annotation and gene expression, but to our knowledge has not been applied to create an organism-wide gene expression atlas [15,18-23].

In this report, we apply RNA-Seq to investigate seven tissues and seven stages in seed development in *G. max*, and compare transcript reads to the most recent release of the *G. max *genome sequence (assembly Glyma1.01). We present an overview of the RNA-Seq data for soybean as a potential model for future RNA-Seq atlases, and address several challenges that arise due to the nature and quantity of next-generation transcriptomic sequence data.

## Results

### Mapping of short-read sequences

Tissues from leaf, flower, pod, two stages of pod-shell, root, nodule and seven stages of seed development were collected from soybean plants (experimental line A81-356022) and raised in growth chambers designed to mimic Illinois field growth conditions. Throughout this manuscript, tissues from stages of development are labeled according to approximated Days After Flowering (DAF) where appropriate (see Experimental Procedures). Total RNA from each tissue and developmental stage was isolated and sent to the National Center for Genome Resources (NCGR) for sequencing. Data from the Illumina Genome Analyzer II instruments produced 5.8 to 8.9 million 36-bp reads for each of seven non-seed tissues and 2.7 to 9.6 million 36-bp reads for each of seven stages of seed development (Additional file 1). The alignment program GSNAP [24] was used to map the reads to two reference genomes: *G. max *and *Bradyrhizobium japonicum*. A digital gene expression analysis was performed on the 'uniquely mappable' genome [15] which includes reads that mapped to the reference genomes with at most two mismatches or one indel and no mismatches [25]. Reads that failed these criteria or mapped to multiple locations were excluded.

The following groups of short-read sequences (from all 14 tissues) were excluded: 14.5% of the reads failed our criteria due to mismatches or indels, 35.2% mapped to multiple locations and 0.2% mapped to the *B. japonicum *genome. Highly repetitive sequences, defined as reads that mapped to 100 or more locations, ranged from 3.6% of the total reads in nodule to 52.3% of the total reads in seed 28-DAF suggesting that these highly repetitive reads may have important functional roles in specific tissues. Further investigation of highly duplicated genes plus transposable elements [26] may be warranted to determine what functional role highly repetitive sequences may have in these tissues.

There were 50.1% of the reads that passed the filtering criteria and mapped uniquely to the *G. max *reference genome. These reads were used in the digital gene expression analysis of all 14 tissues. Of the 66,210 predicted gene models in *G. max *(consisting of 46,430 high-confidence models and 19,780 lower-confidence models), 49,151 (74.2%) genes were transcriptionally active by the following definition: having a sum of at least two counts in one or more tissues in this study (Additional file 2). In the Glyma1.01 annotation set [6], 46,430 genes were identified as "high confidence" as determined by the following criteria: correlation to full-length cDNAs, Expressed Sequence Tags, homology, and *ab initio *methods [12, Supplementary Information section 2]. Of those 46,430 highly-confident genes [6], 41,975 (90.4%) genes were transcriptionally active in this study (Additional file 3) and 4,455 (9.6%) highly-confident genes were not. Conversely, there are an additional 7,176 (10.8%) transcriptionally active gene models from the lower-confidence gene models (Additional file 4). These gene models will be evaluated for possible inclusion to the list of highly-confident gene models.

### Expression and gene structure

Since transcription of genes may be inversely correlated with gene size in plants [27], the coding regions of the predicted genes were inspected to provide insight into the characteristics of an expressed gene in *G. max*. The average lengths of the first, internal and last exon for the predicted genes were 313.6 ± 386.4, 179.8 ± 172.3 and 245.5 ± 298.8 base pairs respectively (Table 1: a). This is similar to values reported for *Arabidopsis: *320.3 ± 371.8, 167.6 ± 195.7 and 328.3 ± 354.5 base pairs, respectively [28]. The GC content of the exons in *G. max *and *A. thaliana *were also similar (Table 1: a). The significance in the size differences were evaluated using the non-parametric Mann-Whitney U test with a p-value cutoff of 10e^-04 ^[27]. Interestingly, the coding regions of genes that were not found to be transcriptionally active in this study were smaller and had a lower GC content than coding regions in genes that were transcriptionally active (Table 1: b,c). A similar trend was found for the genes identified as highly-confident and for genes that were not identified as highly-confident (Table 1: d,e). The genes identified as neither highly-confident nor transcriptionally active (Table 1: f) have the smallest exon length and lowest percentage GC content in *G. max*. This group, which has the smallest genes, may correspond to pseudogenes or may indicate that further improvement is needed in gene model prediction. However, a larger sampling of tissues over several developmental stages and environmental stresses is required before pseudogene determinations can be made.

**Table 1:** Gene structure and transcriptional activity

	first exon	%GC	internal exons	%GC	last exon	%GC	# of exons	total exon length	# of genes
a) All	314 ± 386	44% ± 8%	180 ± 172	42% ± 5%	246 ± 299	44% ± 8%	5.0 ± 5.1	1006 ± 987	66210

b) Expressed	351 ± 418	45% ± 8%	190 ± 178	43% ± 4%	267 ± 315	45% ± 8%	5.5 ± 5.6	1164 ± 1036	49151

c) Not Expressed	207 ± 247	41% ± 8%	141 ± 142	40% ± 6%	170 ± 218	40% ± 7%	3.4 ± 2.8	550.0 ± 638.8	17059

d) Confident	374 ± 438	45% ± 8%	197 ± 185	43% ± 4%	279 ± 328	45% ±7%	5.8 ± 5.7	1263 ± 1062	46430

e) Not Confident	172 ± 141	41% ± 8%	114 ± 77	40% ± 7%	135 ± 117	41% ± 8%	3.0 ± 2.5	403 ± 308	19780

f) Not Expressed, Not Confident	165 ± 129	40% ± 8%	110 ± 73	39% ± 7%	132 ± 108	40% ± 7%	3.0 ± 2.5	395 ± 283	12604

a) All - All 66,210 predicted genes.

b) Expressed - The subset of the predicted genes that were transcriptionally active according to our definition: total of two counts in one or more tissues.

c) Not Expressed - The subset of the predicted gens that were not transcriptionally active according to our definition.

d) Confident - The subset of genes that were identified in the final draft of the soybean genome as highly-confident.

e) Not Confident - The subset of genes that were not identified in the final draft of the soybean genome as highly-confident.

f) Not Expressed, Not Confident - The subset of genes that comprise the intersection of c and d: the genes are neither expressed or highly-confident.

Relationship between exon length, percent GC content and transcriptional activity for three classes of exons: the first exon (first), the internal exons (internal) and last exon (last). The total number of genes in each group and the total exon length is also indicated.

### Tissue-specific analysis of the soybean transcriptome

**Figure 1:**
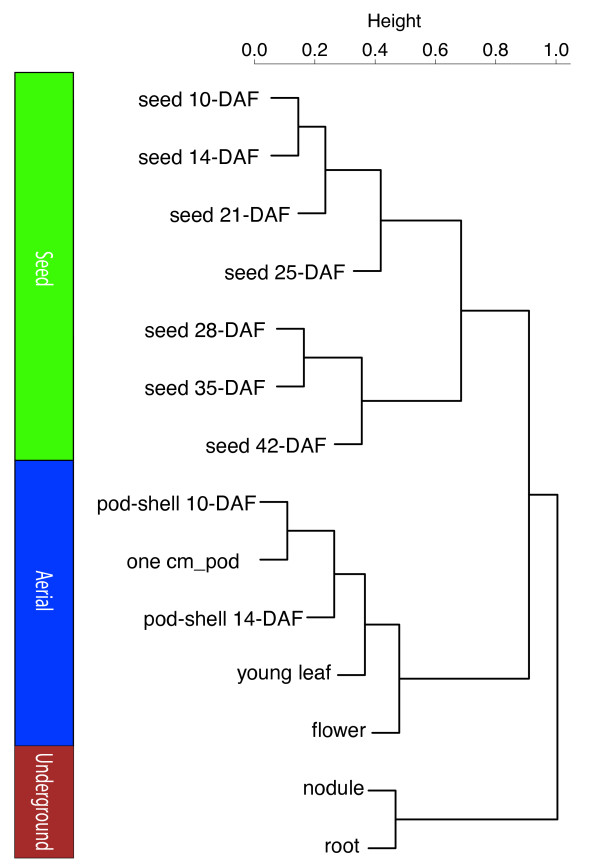
**Hierarchical clustering of transcriptional profiles in 14 tissues**. Hierarchical clustering analysis of the transcriptional profiles was performed using the hclust command in R [39] and the default complete linkage method. The analysis reveals three clades of tissues with similar transcriptional profiles: underground, aerial and seed.

For the tissue-specific analyses, raw digital gene expression counts were normalized using a variation of the reads/Kb/Million (RPKM) method [15,17]. The RPKM method corrects for biases in total gene exon size and normalizes for the total short read sequences obtained in each tissue library. A hierarchical clustering analysis of the transcriptional profiles between tissues and developmental stages using a Pearson correlation suggested three groupings of tissues: underground tissues (root and nodule), seed development (seed 10-DAF, seed 14-DAF, seed 21-DAF, seed 25-DAF, seed 28-DAF, seed 35-DAF and seed 42-DAF) and aerial tissues (leaf, flower, pod-shell 10-DAF, pod-shell 14-DAF and one-cm pod) (Figure 1) [11].

**Figure 2:**
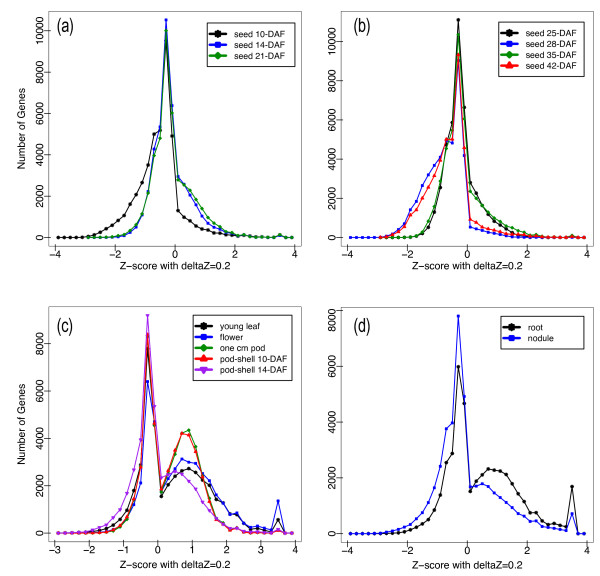
**Relative expression levels based on Z-score analysis**. (a) Relative expression levels in early seed development stages (seed 10-DAF, seed 14-DAF and seed 21-DAF, (b) late seed development stages (seed 25-DAF, seed 28-DAF, seed 35-DAF and seed 42-DAF, (c) aerial tissues (leaf, flower, pod, pod-shell 10-DAF and pod-shell 14-DAF, and (d) underground tissues (root and nodule) were visualized using a Z-score plot. High Z-score values indicate genes with tissue specificity.

A Z-score analysis was conducted to gain insight into the gene expression patterns of each tissue [11]. The Z-score numerical value is calculated by taking for each gene and tissue the (RPKM)-normalized log_2_-transformed transcript count, subtracting the normalized mean transcript count of all tissues and dividing by the standard deviation of the normalized transcript count of all tissues. The Z-score numerical value measures the number of standard deviations the expression level of a gene in a specific tissue is from the mean expression level in all tissues. The Z-score analysis revealed that aerial and underground tissues are distinguished from seed tissue by a bimodal expression pattern with more genes from aerial and underground tissues shifted toward higher expression values (Figure 2). Transcription values in non-seed tissues are less similar than transcription values in stages of seed development resulting in a greater distribution of Z-score values and a noticeable portion of genes with Z-score values near the positive extreme between 3.4 and 3.6 indicating a high specificity for the tissue. We provide a supplementary list of genes with Z-scores in the 3.4 to 3.6 value range for each tissue (Additional file 5). To examine the validity of tissue specificity using Z-score analysis, we inspected the gene annotations based on the Dana Farber tentative consensus sequences [29] for all genes greater than 5000 (RPKM) normalized count in nodule tissues. Of the ten genes with this level of expression found between a Z-score value of 3.4 and 3.6 in nodules, all genes had an annotation. We identified four genes: Glyma10g34290, Glyma10g34280, Glyma20g33290 and Glyma10g34260 as leghemoglobin A, leghemoglobin C1, leghemoglobin C2 and leghemoglobin C3, respectively. We identified another five genes: *Glyma13g40400*, *Glyma14g05690*, *Glyma15g05010*, *Glyma19g22210 *and *Glyma13g44100 *as nodulin 20, nodulin 22, nodulin 24, nodulin 26B and nodulin 44, respectively. The last gene, *Glyma08g14020*, was identified as a nodule specific extensin gene based on the PANTHER classification system [30]. These gene annotations support the validity of using high Z-score values for identifying tissue specific genes from RNA-Seq data.

**Figure 3:**
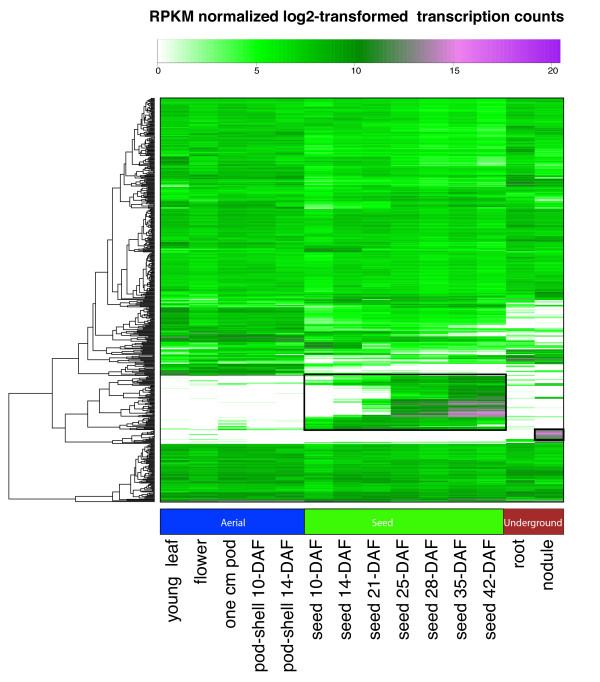
**Heatmap of the top 500 highest expressed genes**. The color key represents RPKM normalized log_2 _transformed counts. Violet indicates high expression, green indicates intermediate expression and white indicates no expression. It is straightforward to identify highly expressed genes in specific tissues from this figure. Tissues are labeled with Days After Flowering (DAF) where appropriate.

A heatmap of the RPKM normalized log_2_-transformed transcription count was generated on the top 500 genes with the highest expression (Figure 3). Two areas on the heatmap indicate high gene expression and specificity to seed and nodule, respectively (Figure 3 boxes). The genes that are specific to nodule are many of the same as those identified by the Z-score analysis (Additional file 6) whereas the genes specific to all of seed development were less apparent in the Z-score analysis (Additional file 7). These genes specific to seed development have gene annotations based on the Dana Farber tentative consensus sequence that include many well known seed specific molecular functions: beta-conglycinin, oleosin, lectin, lipoxygenase, sucrose-binding protein and seed coat BURP domain protein. The high expression levels of these genes suggest an important role during seed development and warrant further investigation especially for those genes with no known annotation. The Z-score analysis of all tissues and heatmap of genes with the highest expression values in this RNA-Seq atlas is provided for further investigation into tissue specific genes.

### Gene specific analysis of transcription in multiple tissues

On the other side of the gene expression spectrum are genes that have little variation across all tissues and developmental stages. These are thought to fulfill housekeeping functions. Housekeeping genes (HKGs) are commonly used as reference genes to normalize expression counts across tissues and developmental stages [31]. As a starting point for identifying HKGs in soybean, we provide a list of 1000 genes generated from the lowest coefficient of variation (CV = standard deviation/mean) among the RPKM normalized expression counts of the predicted gene models in the 14 tissues (Additional file 8).

A GOslim analysis [8,32] on the HKGs was performed to determine what functions are represented in this list. A Fisher's exact test [33] determined the GOslim functions that were over-represented in the HKGs when compared with all expressed genes and indicated an over-representation of the following functions: 4-alpha-glucanotransferase activity (GO:0004134), RNA binding (GO:0003723), mRNA 3'-UTR binding (GO:0003730), structural constituent of ribosome (GO:0003735) and translation initiation factor activity (GO:0003743). Several other organisms contain genes with similar functions have also been indicated as stably expressed adding support to the list of HKGs generated here [12,34].

Interestingly, when all the tissues are included in the HKG analysis only three genes had a correlation of variance below 20%. However, if only the subset of tissues that represent seed development are included in the analysis 324 genes, many of which have HKG related annotations, have a correlation of variance value below 20% (Additional file 9). Although it would be advantageous to identify genes to use as universal references for normalization, it may not be possible to identify genes that are constitutively expressed at high and stable levels in all tissues and developmental stages under all biotic and abiotic stresses. Thus, these lists should be used as guides and the raw (Additional file 10) and RPKM normalized data (Additional file 11) is provided for reanalysis to identify the best constitutively expressed genes in the particular tissues of interest.

**Figure 4:**
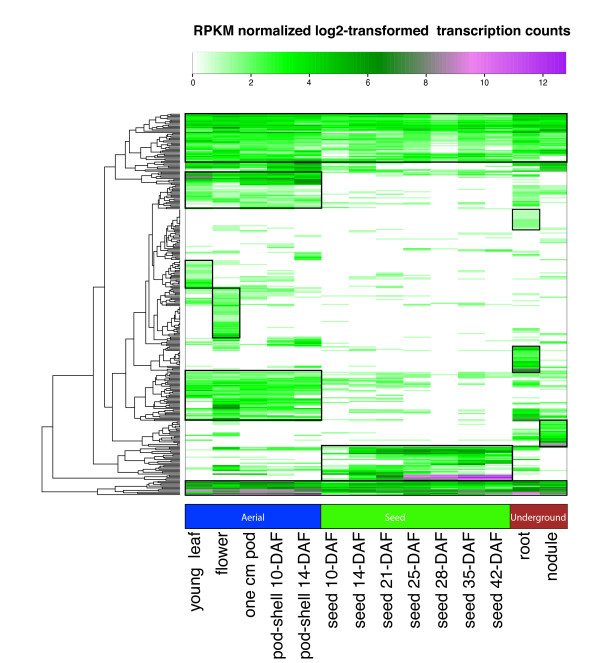
**Heatmap of the Legume Specific Genes**. The color key represents RPKM normalized log_2 _transformed counts of 315 legume specific genes. Violet indicates high expression, green indicates intermediate expression and white indicates no expression. The heatmap suggests some legume specific genes have tissue specific transcription. Tissues are labeled with Days After Flowering (DAF) as appropriate.

In the Glyma1.01 gene set [6], 448 soybean genes were identified as specific to legumes. In the context of the Schmutz et al. paper, this means the gene was identified in *M. truncatula *and *G. max *and not in *Populus trichocarpa *or more distantly-related species. In our study, 315 of these Legume-Specific Genes (LSGs) had a RPKM normalized log_2 _transformed transcription count greater than zero in at least one tissue. An analysis of the transcription patterns of these LSGs in the 14 tissues indicated a propensity for the LSGs to be transcribed in specific tissues as indicated by boxes in the heatmap (Figure 4). Every major tissue group contained a cluster of genes with unique transcription of legume-specific genes. There were also constitutively expressed LSGs with transcription in all tissues. Legume-specific expression in specific tissues is also supported by evidence of preferential gene expression in nodules of *Medicago *[11].

An inspection of the legume specific genes found only in seed development revealed three genes with similar expression profiles that vary between no expression in seed 10-DAF and some of the highest expression seen in the heatmap in seed 42-DAF. The first two genes, *Glyma06g08290 *and *Glyma04g08220 *are oleosins (based on annotations in the Dana Farber tentative consensus sequences). The third gene, *Glyma02g01590*, has an annotated of "lectin precursor 1" [35]. These three genes were also identified in the heatmap for the highest expressed genes. Oleosins are membrane proteins found in seed oil bodies [36] whereas lectin precursor 1 is localized to protein storage vacuoles [35]. A more in-depth analysis may be warranted for these genes to determine how their similar expression profiles in seed development is affected by the negative correlation between protein and oil seed content.

**Figure 5:**
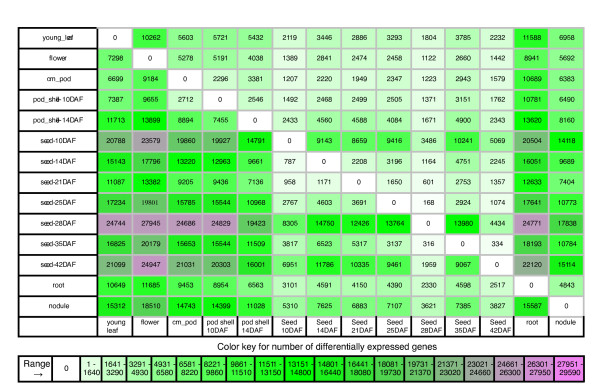
**Tissue by tissue comparison**. This figure shows the total number of genes with a significant increase in gene expression between the row tissue (left) and the column tissue (bottom). For the genes reported in each cell, there is more transcriptional activity in the column tissue than the row tissue. For example, there are 7,298 genes that have a higher transcriptional activity in young leaf than in flower. Also, there are 10,262 genes that have a higher transcriptional activity in flower than in young leaf. These two statements are mutually exclusive and therefore each cell represents a different set of genes.

General trends in expression profiles for all genes were examined by a comparison of the transcription count for every tissue to every other tissue using a Fisher's exact test with a FDR correction of 0.05 [37]. To visualize the number of genes that have significantly different expression between two tissues, we created a table in which each cell represents the number of genes that have a significant increase in gene expression between a tissue on the vertical axis and a tissue on the horizontal axis (Figure 5). Under this scheme, all differentially expressed genes for two tissues, for example root and leaf, are given by the genes in the root (left) to leaf (bottom) cell plus the genes in the leaf (left) to root (bottom) cell. This table indicates that the two tissues with the greatest number of genes exhibiting a significant increase in gene expression occur between seed 28-DAF and flower with 27,945 differentially expressed genes. Similarly, the tissues that have the least number of genes with a significant increase in gene expression occur between seed 25-DAF and seed 28-DAF with 168 genes.

One application of this table is to explore the differential gene expression between two developmental time points in a tissue of interest to gain insight into the gene functions and thereby the biological processes that occur during particular stages of development. For instance, the GOslim molecular functions over-represented in the 168 genes that show a significant increase in gene expression between seed 25-DAF and seed 28-DAF are cellulose synthase activity, nutrient reservoir activity and urease activity. For the 334 genes with a significant increase in gene expression between seed 35-DAF and seed 42-DAF the over-represented GOslim molecular functions are structural constituents of cell wall, nutrient reservoir activity and urease activity. We see that both nutrient reservoir activity and urease activity are important biological processes that occur during these stages of development. A web interface for this table is provided that links each table cell to a downloadable list of genes.

We also explored gene expression in the underground, seed and aerial tissue groups identified by the hierarchical clustering analysis. A gene is considered preferentially expressed in one of these groups if there is a significant increase in transcriptional activity based on a Fisher's exact test (as described above) in at least one tissue of the group over all other tissues. The underground, seed and aerial gene lists contain 6,939 (Additional file 12), 2,036 (Additional file 13) and 6,425 (Additional file 14) genes, respectively.

The characterization of the coding region of genes found in each tissue group may improve our understanding about how exon size and tissue-specificity may be related.

An analysis of exon length and GC content for each of these groups revealed that gene expression in each group of tissues has larger coding regions and a higher percentage of GC content than the average of all predicted genes (Table 2). However, interpretation of these results needs to be made with caution since the larger exon size and higher GC content may be an artifact of identifying significantly expressed genes resulting in an increase in the average transcriptional activity within the group [27]. However, since the process of identifying genes with preferential expression is identical for each group, a comparison between groups is straightforward. Using a Mann-Whitney test [38] to verify our observations, we find that genes with preferential expression for underground tissues have a larger first exon than seed and aerial tissues. On the other hand, aerial tissues have a greater number of exons than seed and underground tissues. The Mann-Whitney test also suggests the differences between the lengths of the total transcribed regions for seed, underground and aerial tissues are significant. The total length of the transcribed regions for each of the tissue groups are 2992.7 ± 4804.5, 3483.8 ± 2738 and 4208 ± 3308.5, respectively. Since the total exon length for each group did not vary significantly, this suggests the average total intron length varies depending on tissues type. Additionally, no significant relationship between GC content and tissue-specificity was found.

**Table 2:** Genes structure and tissue specific gene expression

	first exon	%GC	internal exons	%GC	last exon	%GC	# of exons	total exon length	# of genes
aerial	344.5 ± 392.0	45.9% ± 7.2%	187.5 ± 170.7	43.4% ± 3.7%	267.6 ± 285.9	45.7% ± 7.1%	6.5 ± 5.5	1297.9 ± 882.6	6425.0

seed	353.6 ± 422.4	45.4% ± 7.5%	190.7 ± 178.4	44.0% ± 4.3%	268.4 ± 326.9	45.1% ± 7.5%	4.9 ± 4.7	1097.2 ± 878.7	2036.0

underground	403.4 ± 391.1	46.4% ± 7.4%	204.7 ± 134.9	43.8% ± 4.6%	305.4 ± 309.6	45.9% ± 7.5%	5.1 ± 9.9	1218.9 ± 1954.3	6939.0

aerial - The subset of genes with preferential gene expression in aerial tissues.

seed - The subset of genes with preferential gene expression in seed developmental stages.

underground - The subset of genes with preferential gene expression in underground tissues.

Relationship between exon length, percent GC content and specific tissue groups for three classes of exons: the first exon (first), the internal exons (internal) and last exon (last).

Each list of preferentially expressed genes contains a wealth of information about gene coexpression. As an example, we explored a dendrogram (Additional file 15) generated in the R programming language using the hclust command [39]. A dendrogram shows how genes are clustered based on gene expression but lacks a description of the log_2 _transformed expression data for the genes found in each subclade. Dendrograms of this size were not explored in previous gene expression analyses [10-12] likely due to the challenge of displaying the dendrogram in a meaningful way. The number of genes in the seed dendrogram resulted in a figure that was much wider than it was tall making visualization of the overall clade structure difficult.

**Figure 6:**
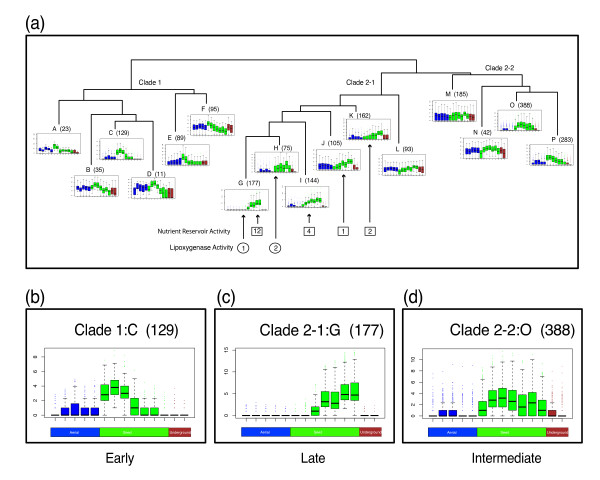
**Boxplot Dendrogram of preferential expressed genes in seed development**. Combination plot of the hierarchical clustering of the genes preferentially expressed in seed stages of development and the RPKM normalized log_2_-transformed expression profiles for the genes in specified subclades represented as a boxplots of each tissue. Boxes contain the number of genes with the GOslim molecular function of nutrient reservoir activity. Circles contain the number of genes with the GOslim molecular function of lipoxygenase activity. Arrows indicate which subclade the specified genes belong. (a) Overview of the clade structure that resulted from the hierarchical clustering analysis is shown. Numbers in parenthesis next to subclades indicates the number of genes represented in the subclade. (b, c, d) Enlarged boxplots of subclades that represent the three main clades defined in the overview as clade 1, clade 2-1 and clade 2-2 are shown. Aerial (leaf, flower, pod, pod-shell 10-DAF and pod-shell 14-DAF), seed (seed 10-DAF, seed 14-DAF, seed 21-DAF, seed 25-DAF, seed 28-DAF, seed 35-DAF, and seed 42-DAF) and underground tissues (root and nodule) are represented in color for each boxplot as blue, green and red respectively.

To better summarize the hierarchical clustering analysis, we present a boxplot dendrogram. The seed dendrogram was simplified to provide the viewer with an overview of the clade structure (Figure 6). The genes below the overview dendrogram, representing a subclade, were grouped together and the log_2 _gene expression values for each tissue in the transcriptional profile were displayed as boxplots (Figure 6). The advantage to this type of display is apparent in the three figure inserts, which indicate that the clustering resulted in three clades. Clade 1, clade 2-1 and clade 2-2 correspond to genes with significant increase in transcription primarily in early seed development (seed 10-DAF, seed 14-DAF and seed 21-DAF), genes with significant increase in transcription primarily in late seed development (seed 25-DAF, seed 28-DAF, seed 35-DAF and seed 42-DAF) and genes with a significant increase in transcription primarily at an intermediate stage in seed development (seed 14-DAF, seed 21-DAF and seed 25-DAF), respectively. Hierarchical clustering was also performed to generate dendrograms for the aerial and underground preferentially expressed genes lists and are provided as supplementary figures (Additional file 16, Additional file 17). All dendrograms and lists can also be access via the RNA-Seq website [40].

## Discussion

In this report we present an RNA-Seq Atlas (Seq-Atlas) for *Glycine max *using next generation Illumina sequencing of the soybean transcriptome. One of the open questions concerning the RNA-Seq method is what to do with short read sequences that map to multiple locations in a genome. This question is particularly relevant in the paleopolyploid genome of *G. max*, which has undergone two rounds of large-scale duplication events in the last ~60 My that resulted in as many as four regions of synteny within most of the genome [6]. Previous studies have indicated the potential for under-representing the total number of counts for a gene especially in closely related gene families [15]. We found that as long as we were aware of the potential pitfalls of under-representing the gene counts, valuable insight into gene expression and the functional relatedness of genes could be obtained from the uniquely mappable reads alone.

Given our limited understanding of the full complexity of the soybean genome, it is gratifying that only a small percentage (3.5%) of the reads that mapped uniquely were located outside the predicted gene models. This suggests that the initial annotation of the soybean genome sequence has captured the majority of transcriptional activity. Using the additional information on transcriptionally active regions, refinement of the existing gene models and the ability to identify new gene models will be improved.

In an analysis of gene-specific expression in multiple tissues, one of the challenges is overcoming the large dynamic range of expression counts generated by next generation sequencing technology to identify genes with similar overall expression profiles. The data presented here has a dynamic range for gene expression greater than six orders of magnitude. Although a log_2_-transformation can significantly reduce the dynamic range, a hierarchical clustering on log_2_-transformed data [11,16,17] has the potential to miss genes with highly similar gene expression profiles but with significantly lower or higher gene expression at each tissue. To identify all genes with similar gene expression profiles, a Fisher's exact test with a FDR correction of 0.05 for a given gene was performed on the raw expression counts between each tissue and every other tissue resulting in a complete description of change in gene expression. Since the Fisher's Exact test normalizes for total counts in the calculation and the comparison was between counts of the same gene and therefore have the same gene length, the raw counts (pre-RPKM normalization) were used. A hierarchical clustering of gene expression based on the direction of change in expression and whether or not it fails the null hypothesis that the expression levels are the same between two tissues identifies all genes with similar expression profiles regardless of the expression levels in each tissue.

In the analysis of tissue-specific gene expression (Figure 1), we determined that the general pattern of gene expression fell into three groups (Figure 1): underground, seed and aerial tissues. The similarity between this clustering using RNA-Seq and the clustering of transcriptionally similar tissues in *Medicago *[11] using Affymetrix GeneChip technology further validates this result. The tissues in soybean are clustered by closely related plant structures: nodules are modified root cortical cells; each seed stage is part of seed development and pods, shells and flowers are modified leaves [41,42]. In addition, seed developmental stages are more similar to aerial tissues than to underground tissues, as seeds are more similar to pods than to roots.

Although expression profile similarity does not necessarily imply similar function, it may provide insight into co-regulated networks of genes. Clusters of genes that are similarly expressed in specific tissues or developmental stages may provide a hint as to the functional role of the genes with no known molecular function. In an effort to divide the data into manageable pieces, we first identified genes that were significantly expressed in seed over the other two tissue groups: underground and aerial. Then, we performed a hierarchical clustering analysis to identify interesting sub-clades of genes with similar expression profiles in seed development. Many of the challenges in displaying and interpreting a dendrogram (Additional file 15) were overcome by combining the dendrogram with log_2_-based boxplots of each tissue (Figure 6) resulted in three clades. Clade 1, clade 2-1 and clade 2-2 contain genes with significant increase in transcription primarily in early, late and intermediate seed development stages. A Fisher's exact test with a Bonferroni correction was performed on the GOslim categories for genes the three clades to determine which GOslim categories were over-represented when compared to the GOslim categories for all genes in the genome. The early seed development clade was over-represented in beta-glucuronidase activity, galactosyltransferase activity, structural constituents of ribosomes and glutamate dehydrogenase activity. The intermediate seed development clade (2-2) was over-represented in leucocyanidin oxygenase activity, whereas the late seed development clade was over-represented in nutrient reservoir activity.

Since seed protein is negatively correlated with seed oil content and yield [43], genes with a GOslim function of nutrient reservoir activity may provide insight into the seed-filling process. To better understand the extent of clustering for genes with nutrient reservoir activity in the late seed development clade and to determine their relationship with seed filling, we identified all genes (143) in *G. max *with a GOslim molecular function corresponding to nutrient reservoir activity (Additional file 18). Of these genes, 83 are transcriptionally active in our data set, with a total transcription count greater than two in all tissues. Of these transcriptionally active genes, 19 are found in four subclades of the late seed development clade (Figure 6a: numbers in squares). Twelve of the genes with nutrient reservoir activity are found in the subclade 2-1:G (Figure 6b). These genes are highly expressed with an RPKM normalized total transcription count in all tissues ranging from 39 to 62,401 counts. Additionally, the genes identified in clade 2-1 with a Goslim molecular function of nutrient reservoir activity are part of the seed-filling process as most of these genes have functions based on the Dana Farber tentative consensus sequence [29,44] that include glycinin, beta-conglycinin and sucrose-binding protein (Additional file 19). Since the other genes in the late seed development clade identified above have similar expression profiles to these 19 genes it is likely that there are other genes in the late seed development clade and in particular, genes in subclade 2-1:G that have similar or complementary roles in seed filling. Further data analysis is required to elucidate how the other genes in the late seed development clade relate to the GOslim-identified nutrient reservoir genes and how insight into the seed filling process will improve seed protein quality, content and yield. This RNA-Seq atlas provides a starting point for such an analysis.

As a final example to demonstrate the power of combining a RNA-Seq atlas with the genomic sequence, consider the soybean lipoxygenase genes (LOXs) [45]. Lipoxygenase enzymes act on polyunsaturated fatty acids to form polyunsaturated fatty acid hydroperoxides that can be converted to aldehydes and alcohols, which result in a lower flavor quality in soybeans [46,47]. Null genotypes were identified in gamma-irradiation experiments that knocked out the three lipoxygenase genes: LOX1, LOX2 and LOX3, that expressed during seed development [48,49]. LOX1 and LOX2 are linked and found on Chromosome 13, while LOX3 is located on Chromosome 15 [45]. The *G. max *Seq-atlas confirms that for the 72 lipoxygenase genes (Additional file 20) identified in the soybean genome and designated with a GOslim molecular function of lipoxygenase activity (GO:0016165), only 3 genes are highly and significantly expressed during seed development based on a Fisher's exact test with a FDR correction of 0.05 during seed development. The genes are: *Glyma13g42310*, *Glyma13g42320 *and *Glyma15g03030 *(Figure 6: numbers in circles). The Seq-Atlas data and the latest genome release support the tight linkage between LOX1 and LOX2 on chromosome 13 - only approximately 7000 base pairs separate the two genes. Although the identities of these lipoxygenase genes were determined prior to knowledge of the genomic sequence and access to next generation sequencing [50], it is not difficult to imagine how the RNA-Seq atlas could be used to increase the efficiency of scientific discovery.

## Conclusions

In summary, the *G. max *Seq-Atlas brings together RNA-Seq data from a diverse collection of tissues and provides new tools for the analysis of large transcriptome data sets obtained from next generation sequencing. This was achieved using the uniquely-mappable short read sequences in an RNA-Seq digital gene expression analysis of the paleopolyploidy soybean genome. We demonstrate how insight can be gained from the global expression patterns of genes, present a method for visualizing a hierarchical clustering of genes based on gene expression and show examples of how this SoySeq-Atlas can be mined. Genomic data from the emerging next generation sequencing technology [50,51] is rapidly accumulating and new methods for analyzing this data is required to improve our understanding of the genetics and genomics of legumes. The SoySeq-Atlas presented here provides a valuable resource for understanding the subtle nuances of the soybean genome and will allow scientists to generate the technological advances in legume agriculture that are required to meet the increasing demand for soybean products.

## Methods

### Plant material and RNA isolation

The seeds were derived from introgressing *G. soja *(PI468916) into *G. max *(A81-356022). Specifically, the BC_5_F_5 _plant P-C609-45-2-2 was heterozygous for the LG I protein QTL introgression from *G. soja*. These seeds were planted directly into pots containing *Bradyrhizobium japonicum*-inoculated soil and supplemented with full nutrient fertilizer (Osmocote 14-14-14) in growth chambers at the University of Minnesota. Chambers were set initially to a photoperiod of 14/10 and thermocycle of 22°C/10°C and monitored to mimic Illinois field growing conditions. Relative humidity settings were 50-60%, and light intensity was measured at 550-740 μE m^-2 ^sec^-1^. All harvests occurred at 1400 hours and consisted of samples pooled from a minimum of three plants [52]. Samples were harvested from plants in parallel and flash frozen in liquid nitrogen before storage at -80°C. Open flowers and young leaf tissue samples were collected simultaneously. Pods and seeds were harvested by seed weight and pod lengths that correspond to approximated Days After Flowering (DAF) as specified. The one-cm pod was processed intact (approximately 7-DAF), while the four and five cm pods (approximately 10-13 DAF and 14-17 DAF) were divided into seed and pod-shell components. Seed 21-DAF, Seed 25-DAF, Seed 28-DAF and Seed 35-DAF had seed weights between 10 and 25 milligrams, 25 and 50 milligrams, 50 and 100 milligrams, 100 and 200 milligrams, and greater than 200 milligrams, respectively.

Root and nodule tissues were harvested from plants grown in growth chambers set to 16-hr photoperiods with light intensities ranging from 310-380 μE m^-2 ^sec^-1^. Seeds were imbibed for three days, planted in quartz sand and fertilized with a full nutrient solution. Root tissue was harvested after 12 days. Nodules were harvested at 20-25 days after inoculation; for these samples, plants were fertilized for the first seven days with nutrient solution containing 3.5 mM NO_3 _and subsequently fertilized every other day with a full nutrient solution lacking nitrogen.

Soybean tissue samples were ground with liquid nitrogen by mortar and pestle. Total RNA was isolated by a modified TRIzol^® ^(Invitrogen) protocol [53]. DNA was removed by digest with on-column RNase-free DNase (Qiagen), and RNA was purified and concentrated by RNeasy column (Qiagen). RNA quality was evaluated by gel electrophoresis, spectrophotometer and Agilent 2100 bioanalyzer.

## Plant Ontology

The plant ontology obtained from Soybase [54] gives an approximate stage of development for each tissue in this study (Table 3). These definitions are based on development stages in tissues as presented by Carlson and Lersten (2004) [55] and Le et. al. (2007) [7]. The developmental process is affected by genotype, temperature, lighting and nutrition. Therefore, the plant ontology is provided as an estimate of the developmental stage of each tissue.

**Table 3:** Plant Ontology

Tissue	DAF	Ontology term	Ontology identifier
Young leaf	NA	0.4 Leaflets unfurled	SOY:0000252

Flower	NA	F0.4 Open flower	SOY:0001277

One cm pod	7 DAF	F0.7 Small size pod	SOY:0001280

Pod-shell	10-13 DAF	F0.8 Pod medium size	SOY:0001281

Pod-shell	14-17 DAF	F0.9 Full pod size	SOY:0001282

Seed	10-13 DAF	S1.06 Cotyledon stage	SOY:0001290

Seed	14-17 DAF	S1.06 Cotyledon stage	SOY:0001290

Seed	21 DAF	S1.07 Early maturity stage 1	SOY:0001291

Seed	25 DAF	S1.07 Early maturity stage 1	SOY:0001291

Seed	28 DAF	S1.07 Early maturity stage 1	SOY:0001291

Seed	35 DAF	S1.08 Early maturity stage 2	SOY:0001292

Seed	42 DAF	S1.09 Mid seed maturity	SOY:0001293

Root	NA	Root structures	SOY:0001183

Nodule	NA	Bacterial root nodule	SOY:0001301

Ontology terms for the 14 tissues in this study. Days After Flowering (DAF) are approximated based on pod length and seed size.

### Exon length and transcriptional activity

A gene is considered transcriptionally active by our definition if two or more short read sequences uniquely map to a gene in one or more tissues. The first, internal, last exon lengths, percentage GC content, number of exons, and total exon length were extracted from the Glyma1.gff file represented on Soybase [56] as the 'gene models' track (Glyma1.01 genome assembly). Internal exon lengths were averaged and standard deviation was calculated for all values.

### Sequencing, data processing, normalization and analysis

Total RNA was sent to the National Center for Genome Resources for next generation Illumina sequencing. Poly-A containing RNA was isolated from total RNA using oligo-dT25 magnetic beads (Dynabeads; Invitrogen, Carlsbad, CA). The resulting RNA is denatured and used as template for random-primed cDNA synthesis then end repaired withT4 DNA polymerase, Klenow polymerase and dNTPs. The polished fragments are phosphorylated by T4 polynucleotide kinase, followed by the addition of a single "A" base to the 3'-end of the blunt-ended phosphorylated DNA fragments. Illumina adapters are then added to the DNA fragments by ligation and size selected by electrophoresis for a desired size range of ~500 bp. Purified DNA libraries are amplified by PCR for 15 cycles. Libraries are qualitatively and quantitatively assessed by Nanodrop ND-1000 (Thermo Scientific, Waltham, MA) UV/Vis spectroscopy and DNA BioAnalyzer 2100 microfluidics (Agilent, Santa Clara, CA). Two picomoles of the size-selected cDNA library are loaded on an Illumina single-end flow cell using the Illumina Cluster Station (Illumina, Inc., San Diego, CA). 36 bp reads are collected on an Illumina Genome Analyzer using sequencing-by-synthesis technology. Image data acquired from the sequencing run were mirrored to an off-instrument computer using the Illumina platform to perform image analysis, base-calling, quality filtering, and per base confidence scores. Sequence reads were then aligned using GSNAP [24] against a reference composed of the 8× soybean genome sequence assembly (Glyma1.01 genome assembly) to which was added the genome sequence of the symbiont *Bradyrhizobium japonicum*. The default settings in GSNAP were used. These settings include spliced alignments of the transcript reads to the genomic reference sequences requiring canonical splice sites and allowing introns of up to 10,000 bp; alignments were also allowed to include small indels and mismatches. The reads were divided into five classes: not mappable reads, highly repetitive reads, rhizobium reads, unique high quality reads, repetitive high quality reads and low quality reads.

A sequence was considered not mappable if a read did not map to an interval on the reference genomes as identified by GSNAP [24]. If the Illumina read sequence mapped to over 100 locations on the reference genomes or to the *B. japonicum *reference genome then the sequences were considered highly repetitive or of rhizobium origin respectively. The remaining Illumina reads were sorted into high quality or low quality reads based on a cutoff of no more than two mismatches or one indel and no mismatches [25]. The high quality reads were further subdivided into uniquely mappable reads and repetitive reads if the best mapping for the read matched only one location or if the read mapped to 2-100 locations respectively. Reads from the high quality category that mapped uniquely were used for the digital gene expression counts. The determination of digital gene expression counts for each soybean gene model was performed in R (Additional file 21). The boundaries of each gene were taken as the maximal starting and ending positions from any of the transcripts associated with the gene, and any read alignment partially contained within this span was counted toward the expression of that gene in the given sample. The raw digital gene expression counts were normalized using a slight variation of the RPKM method [15,17]. The following equation was used: RPKM = 10^9^(C)/(N L) where C is the uniquely mapped counts determined from the high quality category, L is the length of the cDNA for the longest splice variant for a particular gene model and N is the total mappable reads which was determined as the sum of the high quality reads and the highly repetitive reads. Log_2_-transformations of this normalization were performed as specified in the methods below.

### Gene list generation and Hierarchical clustering

Transcriptionally active genes were identified as genes with at least two uniquely mapped raw counts in any combination of tissues or developmental stages. The Fisher's Exact test with a false discovery rate correction of 0.05 [37] was performed on every combination of the 14 tissues resulting in a 196-element vector of change in transcriptional activity for each gene. Each element of the vector was assigned a -1, 0 or 1 corresponding to a significant decrease, no change, or a significant increase in transcriptional activity respectively. To identify genes with preferential gene expression in one of the underground, seed or aerial tissue groups (Figure 6), the 196-element vector generated using the Fisher's exact test was filtered for genes that showed a significant increase in transcriptional activity in at least one tissue of the group over all tissues not in the group. Hierarchical clustering of genes in these lists was used to generate dendrograms for each tissue group based on the 196-element vector using the hclust command and the default complete linkage method in R [39]. To generate the boxplot dendrogram, nodes were chosen that provided an overall picture of the clade structure. Genes below these nodes were grouped into subclades and a boxplot analysis was performed on the RPKM normalized log_2_-transformed data for the 14 tissues. The dendrograms and subclade boxplots generated in R were manually combined in Adobe Illustrator.

The tissue by tissue comparison of change in transcriptional activity was generated from the 66,210 row by 196 column matrix. There are 66,210 genes and 196 combinations of possible changes in transcriptional activity. A column sum was performed on the 66,210 rows for which there was a significant increase in transcriptional activity. The resulting 196 vector of column sums was then reshaped into a 14 by 14 tissue by tissue comparison.

### GOslim analysis

The 66,210 predicted gene sequences in *G. max *(Glyma1.01 genome assembly) were compared with the predicted genes in the *Arabidopsis *genome (TAIR v. 8) [57] using TBLASTX (E < 10^-6^, [58]. The *Arabidopsis *gene model of estimated best fit was then connected to the concurrent gene ontology [32]. The annotations of the *Arabidopsis *gene model that best fit each soybean gene model were used as the basis for our gene ontologies.

A GOslim analysis was performed to determine over-representation of molecular function in selected groups. The number of genes connected to each GOslim category was counted for both the population and specified group. Then the Fisher's exact test was performed on each individual GOslim category found in the specified group [8]. A Bonferroni adjustment [59] of P-values was made to correct for over sampling. The P-value from the Fisher's exact test for each GOslim category was multiplied by the total number of Goslim categories in the specified group. Those GOslim categories with a P-value less than 0.05 after the Bonferroni correction were considered significantly over-represented.

### Heatmap generation

A heatmap of the legume-specific genes and the 500 highest expressed genes was generated in R using the heatmap.2 function in the gplots CRAN library. The legume-specific genes that did not have a RPKM normalized log_2_-transformed transcription count greater than zero in at least one tissue were excluded leaving 315 genes. The LSGs were taken from the Glyma1.01 gene set [6]. The highest expressed genes were determined based on the sum of raw counts in all tissues. Boxes were added to indicate clusters of genes that are similarly expressed in specific tissues. Supplementary figures (Additional file 22 and Additional file 23) are supplied with additional detail indicating the gene represented by each cell in the heatmap.

### Z-score

Calculation of the Z-score was determined based on the RPKM-normalized log_2_-transformed transcript count data as follows: Z = (X-μ)/σ, where X is the transcript count of a gene for a specific tissue/timepoint, μ is the mean transcript count of a gene across all tissues/developmental stages and σ is the transcript count standard deviation of a gene across all tissues/developmental stages. All calculations and plotting were performed in R [39].

## Abbreviations

RPKM: Reads/Kb/Million; DAF: Days After Flowering; GO: Gene Ontology; LSG: Legume Specific Genes; HKG: House Keeping Genes; LOX: Lipoxygenase gene; mya: millions of years ago; RNA-Seq: RNA Sequencing; NCGR: National Center for Genome Resources.

## Authors' contributions

AJS and JLW conceived the analyses; JLW performed the GOslim analyses with MG. AJS conducted the analyses and prepared the manuscript. CPV and RCS provided advice on seed development staging. YB provided and prepared the total RNA for submission to NCGR for Illumina sequencing by ADF and GDM. SC, DG and RN aided with the analyses and gene ontology. All authors discussed the results and improved the manuscript.

## Supplementary Material

Additional file 1 **Summary of the RNA-Seq short read sequences**. This table includes the number of reads that were of low quality, not mappable to the reference genomes, mapped to the B. Rhizobium genome, highly repetitive (mapped to over 100 locations), repetitive (mapped between 2-100 locations), High quality reads (reads that passed our filtering criteria), unique (mapped to 1 location) and total mappable reads (sum of the highly repetitive and high quality reads). Click here for file

Additional file 2 **Transcriptionally active genes from all predicted gene models**. List of gene models from all the predicted gene models that were transcriptionally active. A gene model was considered transcriptionally active if the sum of the raw counts that mapped to the model in one or more tissues was greater than 1. Click here for file

Additional file 3 **Transcriptionally active genes from the highly-confident gene models**. List of gene models from the highly-confident gene models that were transcriptionally active. A gene model was considered transcriptionally active if the sum of the raw counts that mapped to the model in one or more tissues was greater than 1. Click here for file

Additional file 4 **Transcriptionally active genes not from the highly-confident gene models**. List of gene models that were transcriptionally active but not part of the list of genes models that are currently considered highly-confident gene models. A gene model was considered transcriptionally active if the sum of the raw counts that mapped to the model across all tissues was greater than 1. Click here for file

Additional file 5 **Tissue Specific genes based on Z-score analysis**. List of gene models that are that have Z-score value between 3.4 and 3.6 in each tissue. Click here for file

Additional file 6 **Nodule Specific gene expression**. Genes with high gene expression specific to nodule tissue. Click here for file

Additional file 7 **Seed specific gene expression**. Genes with high gene expression specific to seed development. Click here for file

Additional file 8 **Potential House keeping genes**. The top 1000 gene models that showed the lowest coefficient of variance (CV) among all the predicted gene models for all 14 tissues (CV = standard deviation/mean). Click here for file

Additional file 9 **Housekeeping genes in seed development**. Gene list sorted by coefficient of variance for the seven stages in seed development. Click here for file

Additional file 10 **Raw short read sequence count data**. Raw short read sequence count data after our filtering criteria (see methods) but before normalization for every predicted gene model in 14 tissues. Click here for file

Additional file 11 **RPKM normalized short read sequence count data**. Short read sequence count data after RPKM normalization for every predicted gene model in 14 tissues. Click here for file

Additional file 12 **Genes significantly expressed in underground tissues**. List of gene models for which there was as significant change in gene expression in one of the underground tissues (root and nodule) over all other tissues in this study. Click here for file

Additional file 13 **Genes significantly expressed in seed tissues**. List of gene models for which there was as significant change in gene expression in one of the seed tissues (seed 10-DAF, seed 14-DAF, seed 21-DAF, seed 25-DAF, seed 28-DAF, seed 35-DAF and seed 42-DAF) over all other tissues in this study. Click here for file

Additional file 14 **Genes significantly expressed in aerial tissues**. List of gene models for which there was as significant change in gene expression in one of the aerial tissues (young leaf, flower, one cm pod, pod-shell 10-DAF, pod-shell 14-DAF) over all other tissues in this study. Click here for file

Additional file 15 **Hierarchical clustering of genes significantly expressed in seed tissues**. Hierarchical clustering dendrogram of genes with significant expression in seed tissues. Click here for file

Additional file 16 **Hierarchical clustering of genes significantly expressed in aerial tissues**. Hierarchical clustering dendrogram of genes with significant expression in aerial tissues. Click here for file

Additional file 17 **Hierarchical clustering of genes significantly expressed in underground tissues**. Hierarchical clustering dendrogram of genes with significant expression in underground tissues. Click here for file

Additional file 18 **All genes annotated with nutrient reservoir activity**. List of gene models from all predicted models that have a GOslim annotation of nutrient reservoir activity. Click here for file

Additional file 19 **Genes annotated with nutrient reservoir activity expressed in seed development**. Table of gene models with a GOslim annotation of nutrient reservoir activity found in seed development and their possible function based on their homologous Dana Farber tentative consensus sequence. Click here for file

Additional file 20 **All genes annotated with lipoxygenase activity**. List of gene models from all predicted models that have a GOslim annotation of lipoxygenase activity. Click here for file

Additional file 21 **Interval matching script**. Script to perform interval matching of short read sequence intervals after lignment with GSNAP and predicted gene models (Glyma1.01 genome assembly). Click here for file

Additional file 22 **Heatmap of highest expressed genes**. This figure is the actual output from the heatmap.2 R command. Each cell in heatmap for the highest expressed genes contains the name of the gene model. Click here for file

Additional file 23 **Heatmap of legume specific genes**. This figure is the actual output from the heatmap.2 R command. Each cell in the heatmap for the legume specific genes contains the name of the gene model. Click here for file
