# Supplementary figures and images for: RNA-Seq Atlas of Glycine max: A guide to the soybean transcriptome
Source: BMC Plant Biol. 2010 Aug 5;10:160. doi: 10.1186/1471-2229-10-160 (PMC3017786; doi:10.1186/1471-2229-10-160)

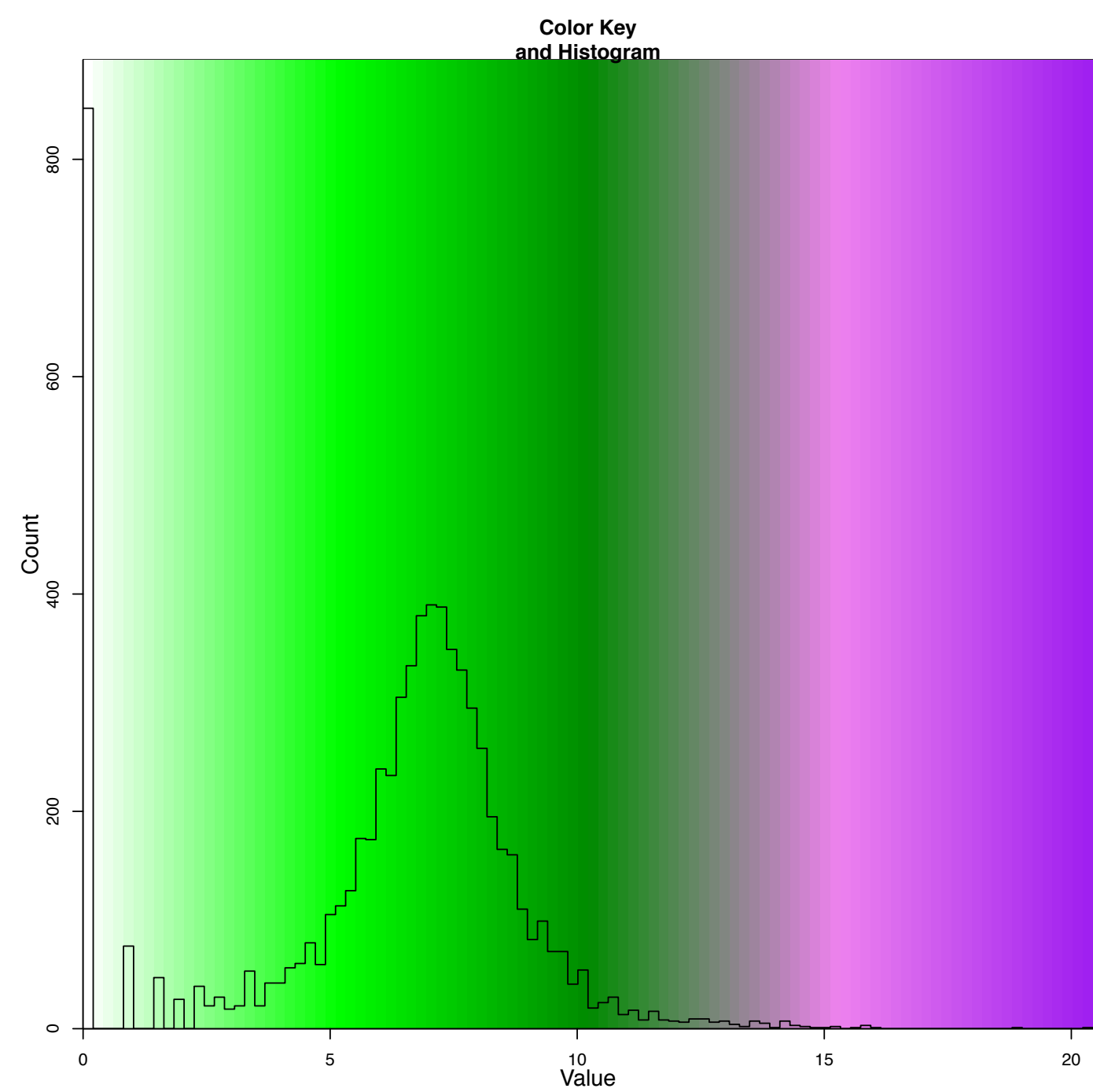

Heatmap of the top 500 highest expressed genes

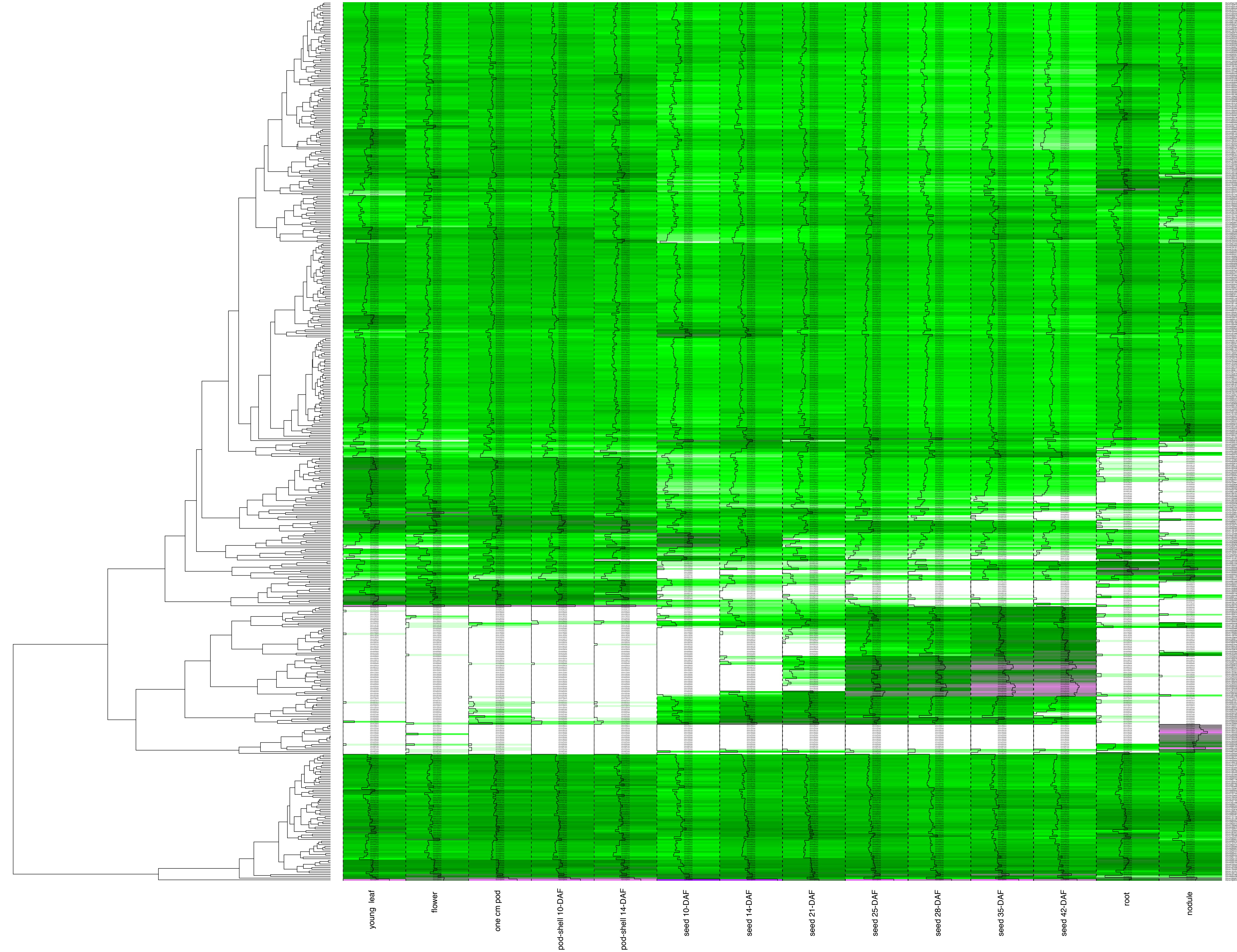

Supplement: Additional file 22 — Heatmap of highest expressed genes. This figure is the actual output from the heatmap.2 R command. Each cell in heatmap for the highest expressed genes contains the name of the gene model. [file 1471-2229-10-160-S22.PDF]

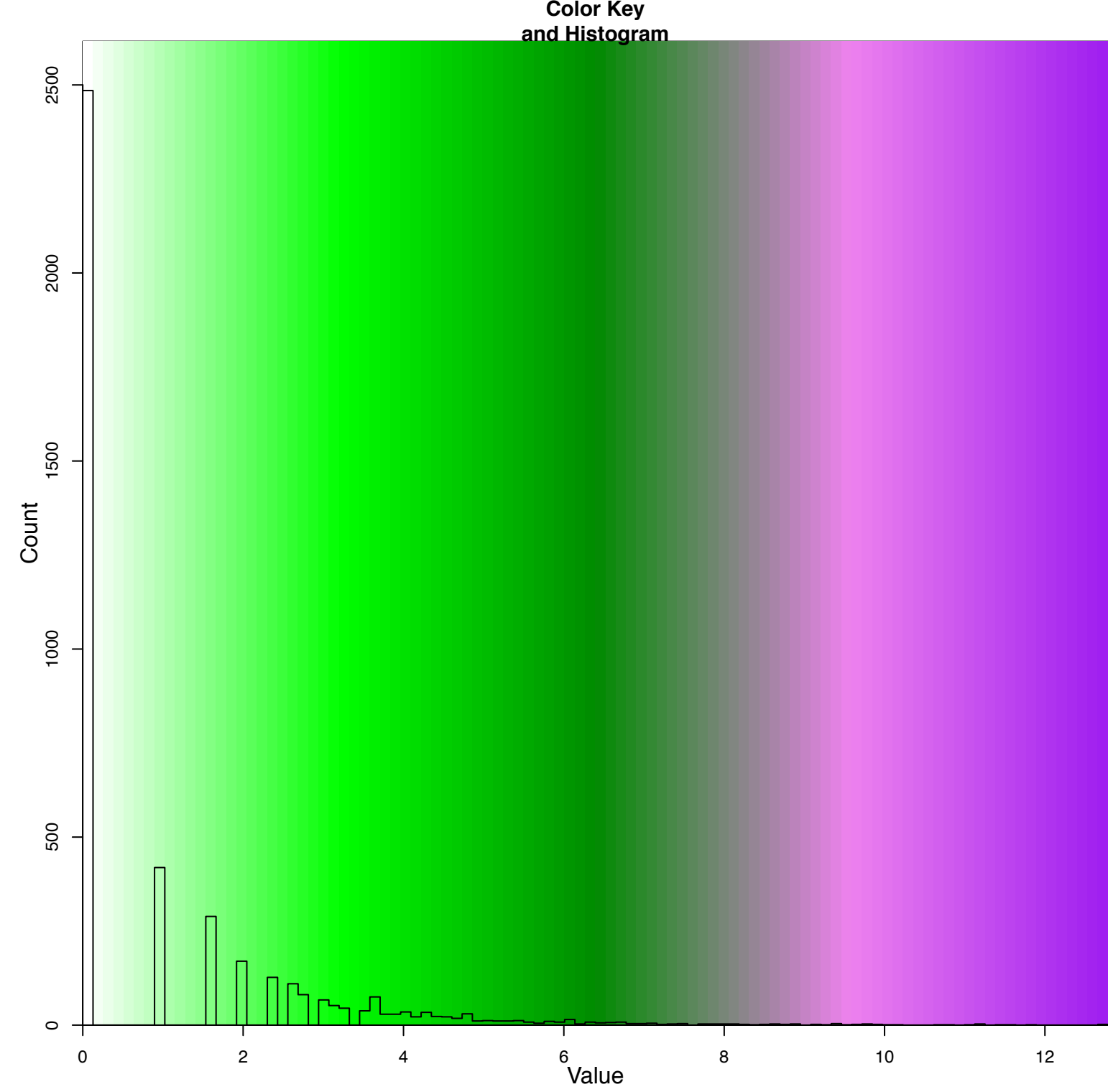

## Heatmap of 315 Legume Specific Genes

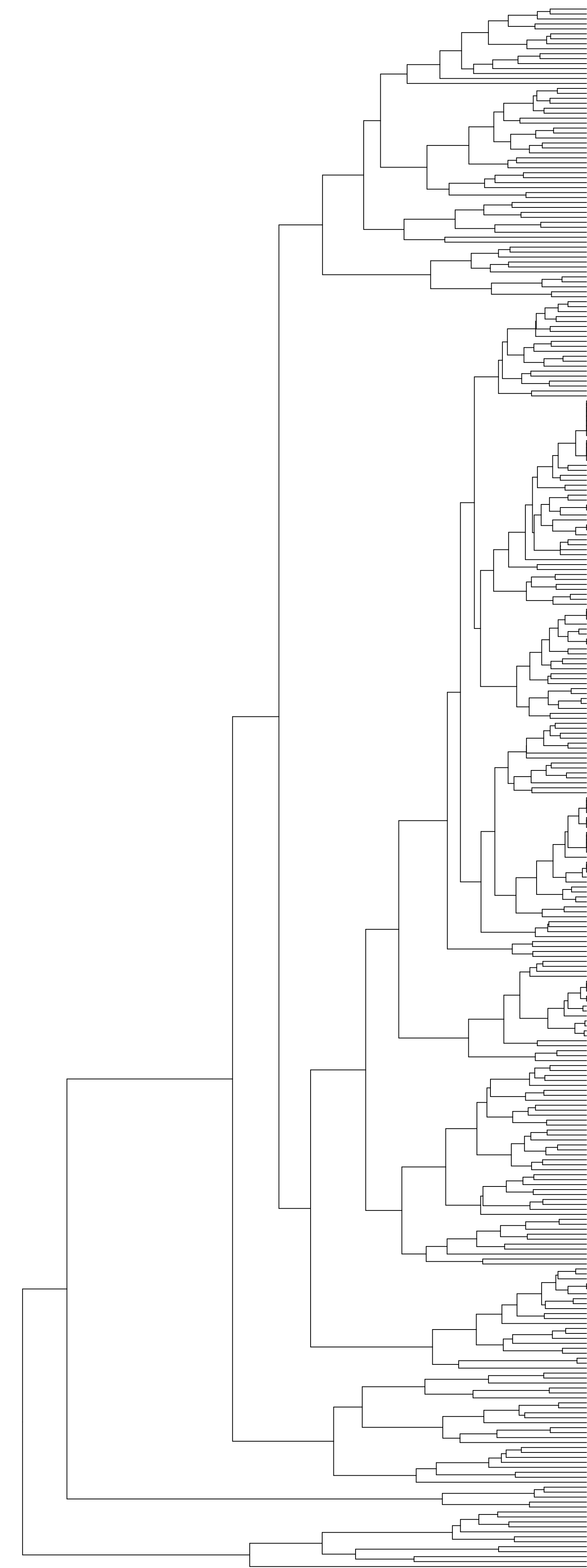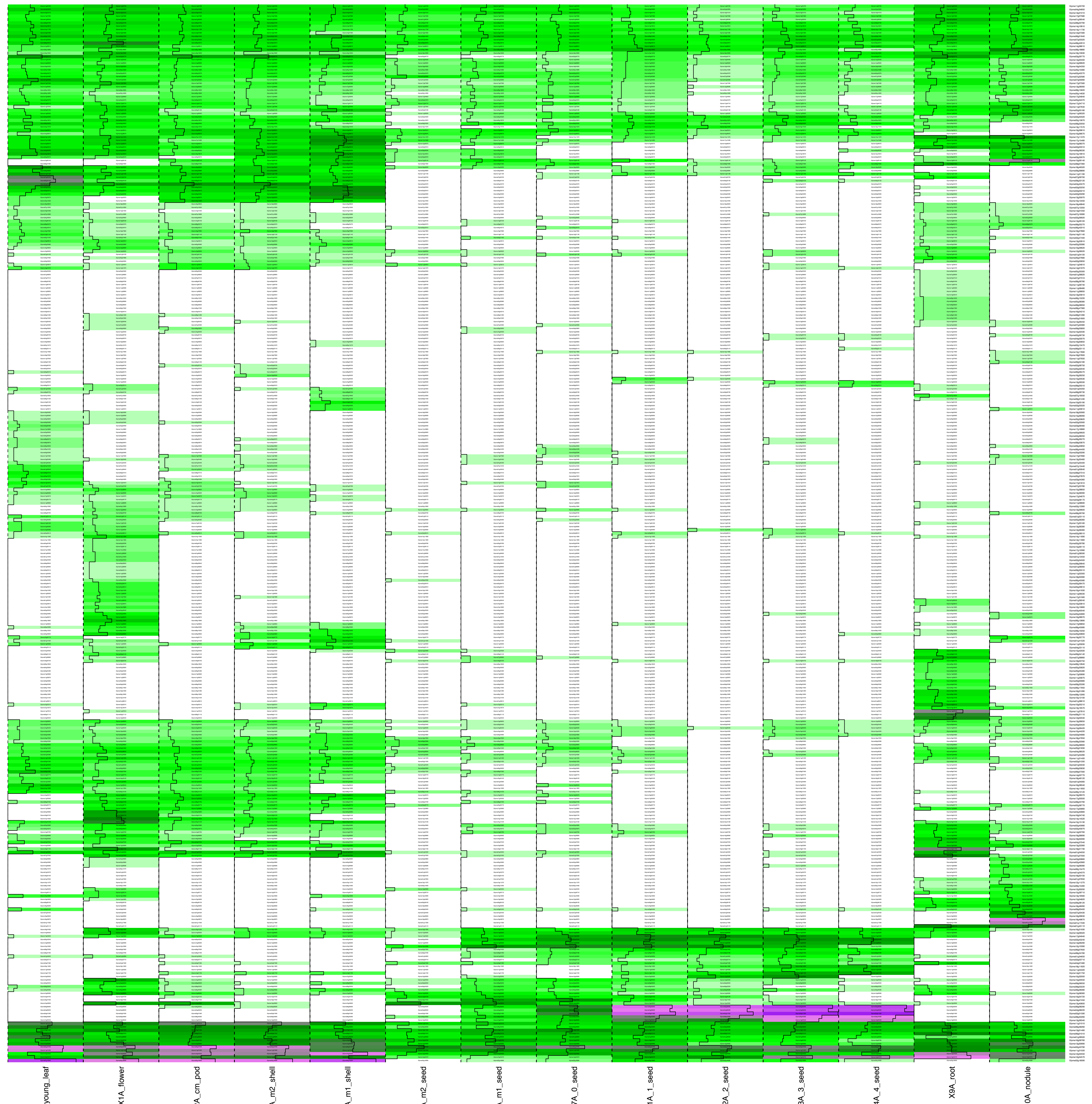

Supplement: Additional file 23 — Heatmap of legume specific genes. This figure is the actual output from the heatmap.2 R command. Each cell in the heatmap for the legume specific genes contains the name of the gene model. [file 1471-2229-10-160-S23.PDF]
