# Supplementary material for: RNA-Seq Atlas of Glycine max: A guide to the soybean transcriptome
Source: BMC Plant Biol. 2010 Aug 5;10:160. doi: 10.1186/1471-2229-10-160 (PMC3017786; doi:10.1186/1471-2229-10-160)

Hierarchical clustering dendrogram of genes with significant expression in seed tissues.

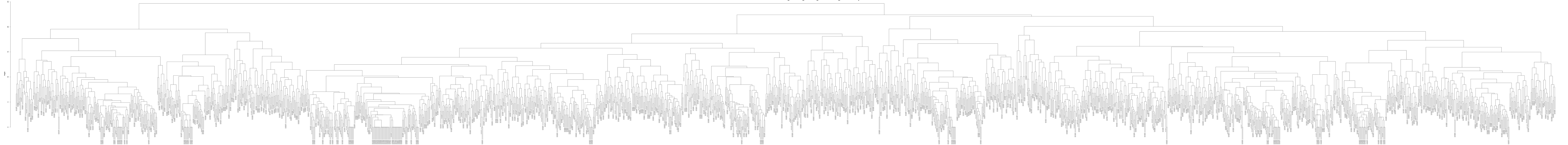

Supplement: Additional file 15 — Hierarchical clustering of genes significantly expressed in seed tissues. Hierarchical clustering dendrogram of genes with significant expression in seed tissues. [file 1471-2229-10-160-S15.PDF]
