# Supplementary material for: RNA-Seq Atlas of Glycine max: A guide to the soybean transcriptome
Source: BMC Plant Biol. 2010 Aug 5;10:160. doi: 10.1186/1471-2229-10-160 (PMC3017786; doi:10.1186/1471-2229-10-160)

Hierarchical Clustering of genes significantly expressed in aerial tissues

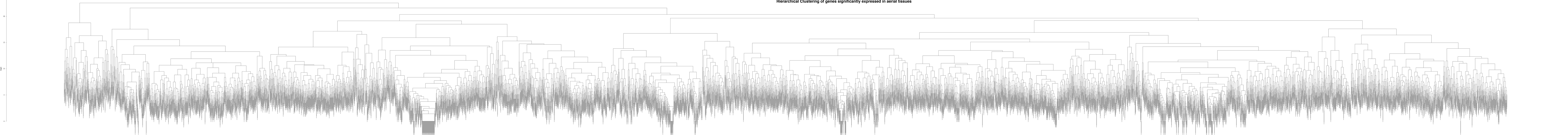

Supplement: Additional file 16 — Hierarchical clustering of genes significantly expressed in aerial tissues. Hierarchical clustering dendrogram of genes with significant expression in aerial tissues. [file 1471-2229-10-160-S16.PDF]
