# Supplementary material for: RNA-Seq Atlas of Glycine max: A guide to the soybean transcriptome
Source: BMC Plant Biol. 2010 Aug 5;10:160. doi: 10.1186/1471-2229-10-160 (PMC3017786; doi:10.1186/1471-2229-10-160)
Supplement: Additional file 17 — Hierarchical clustering of genes significantly expressed in underground tissues. Hierarchical clustering dendrogram of genes with significant expression in underground tissues. [file 1471-2229-10-160-S17.PDF]

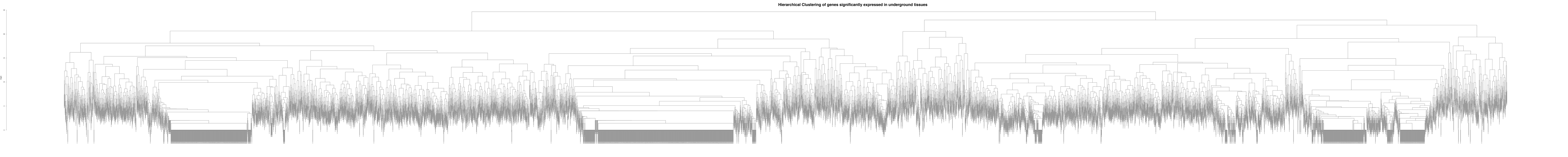
Hierarchical Clustering of genes significantly expressed in underground tissues
Height
0
5
10
15
20
25
